# Mesenchymal Stem Cells Isolated from Equine Hair Follicles Using a Method of Air-Liquid Interface

**DOI:** 10.1007/s12015-023-10619-w

**Published:** 2023-09-21

**Authors:** Hanluo Li, Shiwen Xiong, Federica Francesca Masieri, Seltenhammer Monika, Bernd Lethaus, Vuk Savkovic

**Affiliations:** 1 National “111” Center for Cellular Regulation and Molecular Pharmaceutics, Hubei Provincial Key Laboratory of Industrial Microbiology, Sino-German Biomedical Center, Hubei University of Technology, Wuhan, 430068 Hubei Province China; 2 Department of Cranial Maxillofacial Plastic Surgery, University Clinic Leipzig, 04103 Leipzig, Germany; 3 School of (EAST) Engineering, Arts, Science & Technology, University of Suffolk, Ipswich, IP 41QJ UK; 4 Institute of Livestock Sciences (NUWI), University of Natural Resources and Life Sciences, Vienna, Gregor-Mendel-Straße 33/II, A-1180 Vienna, Austria

**Keywords:** Equine hair follicles, Mesenchymal stem cells, Autologous veterinary therapy, Minimal-invasive cell source, Tri-lineage differentiations

## Abstract

Equine mesenchymal stem cells (MSC) of various origins have been identified in horses, including MSCs from the bone marrow and adipose tissue. However, these stem cell sources are highly invasive in sampling, which thereby limits their clinical application in equine veterinary medicine. This study presents a novel method using an air-liquid interface to isolate stem cells from the hair follicle outer root sheath of the equine forehead skin. These stem cells cultured herewith showed high proliferation and asumed MSC phenotype by expressing MSC positive biomarkers (CD29, CD44 CD90) while not expressing negative markers (CD14, CD34 and CD45). They were capable of differentiating towards chondrogenic, osteogenic and adipogenic lineages, which was comparable with MSCs from adipose tissue. Due to their proliferative phenotype in vitro, MSC-like profile and differentiation capacities, we named them equine mesenchymal stem cells from the hair follicle outer root sheath (eMSCORS). eMSCORS present a promising alternative stem cell source for the equine veterinary medicine.

**Graphical abstract:**

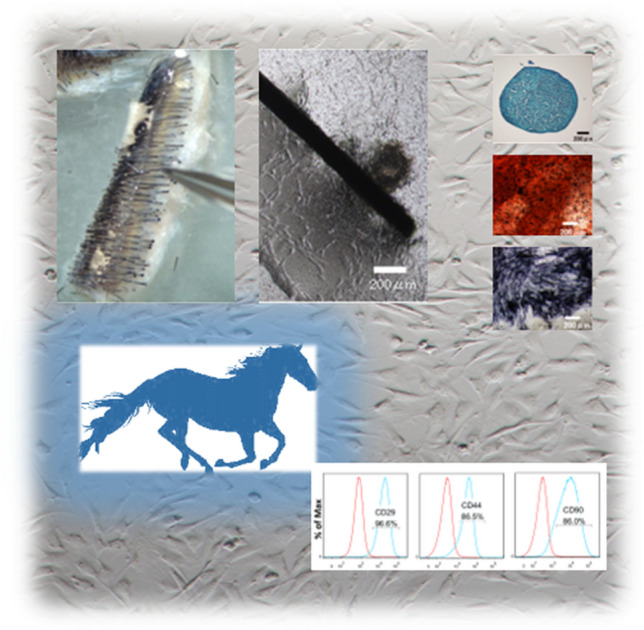

## Introduction

Regenerative cell-based therapies have been employed in horses since early 2000’s as succeeding technologies to the corresponding booming human-designated treatments. Among those, the most promising applications of cell therapy in equine so far have been based on Mesenchymal Stem Cells (MSC) [1–4]. Therapeutic development of MSCs has been moving forward towards application, but remains limited in their clinical impact to date. MSCs generally meet fewer methodological, ethical and regulatory challenges compared to either of their counterparts, especially embryonic stem cells and induced pluripotent stem cells. MSCs are somatic stem cells, easily obtained autologously and their isolation and auto-transplantation create no ethical challenges. MSCs also showed lower risks on forming teratomas or teratocarcinomas after being reinfused to recipients in clinical studies [5].

MSCs are reliably available due to their defined localisation in stem cell niches. To this day, MSCs used for clinical trials in horses have typically been isolated from bone marrow and, to a lesser extent, from adipose tissue or umbilical cord blood, umbilical cord matrix (Wharton’s Jelly), placental tissue, and amniotic fluid [6–12]. The main therapeutic applications of these equine MSCs included primarily horse tendon injuries and osteoarthritis, as well as other cartilage and ligament injuries [1]. The MSC-based treatments brought about improved physiological recovery, earlier re-engagement in sport activities and a lower incidence of re-injury in treated animals [13–16]. For example, based on the regenerative effects of equine umbilical cord MSCs, a veterinary medicine called HorStem has been approved as a medicinal product in the European Union [16]. Another product under a name Arti-Cell Forte, designed as a blood-derived stem cell suspension to be injected into inflamed joints, has been authorised for marketing by the European Medicines Agency (EMA) in 2019 [17, 18]. Although equine MSCs carry a promise as powerful treatments of degenerative diseases or injuries, they still come with multiple disadvantages regarding their availability, invasivity, and lack of a standardized therapeutical effect.

The most reliable method of obtaining MSCs in horses is isolating from the bone marrow [19, 20]. Bone marrow is collected from the sternum or the tuber coxae by a Jamshidi punction needle under sedation, which is often performed in surgical settings using ultrasonography [21, 22]. Equine bone marrow aspirate can be directly plated on the tissue culture plastic to cultivate large numbers of adherent bone marrow-derived mesenchymal stem cells (BM-MSCs), or immediately centrifuged, providing lower cell numbers in bone marrow concentrate (BMC) [14]. Several culture and centrifugation systems for obtaining MSCs from bone marrow are available on the market, routinely used by equine veterinary medicine practitioners [23, 24]. A main downside of BM aspiration is its invasive biopsy sampling, involving not only demanding clinical settings but also substantial pain, general anesthesia, tissue trauma at the puncture site, haematoma, donor site morbidity and a risk of infection [25]. In therapy, BM-MSCs are used to treat intra-articular soft tissue injuries in horses, as well as for cartilage regeneration by enhancing early chondrogenesis and generally improving repair of full-thickness cartilage lesions [13, 26–28]. The outcomes of BM-MSC-based treatment of primary OA were more variable [29, 30].

MSCs derived from adipose tissue appear to exhibit limited matching effects to those of BM-MSCs in bone- and cartilage regeneration. Fat tissue is abundantly available and its digestion retrieves a relatively large portion of nucleated cells. Adipose tissue in horses is either harvested by lipoaspiration with a hypodermal needle or surgically excised by a longitudinal 10 cm long section at the lower back pad region, followed by 5 ml fat tissue collection by resection or curettage [31] Upon harvesting and collagenase digestion, the obtained tissue is either cultured to obtain adipose tissue-derived MSCs (AD-MSCs) or subjected to isolation of the stromal vascular fraction (AD-SVF) cells [18, 31–33]. The latter procedure is quickly available (48 h) without the need for a primary cell culture step. AD-SVF are more popular in clinical use due to their short turnaround time despite containing on average a 20–40% MSC fraction [18] However, upon isolation from lipoaspirate, stromal vascular fraction cells can turn apoptotic and necrotic with uncontrolled release of cellular content at the disrupted donor site, including pro-inflammatory mediators, which may complicate the outcome of the candidate cell therapy [34]. Altogether, therapeutic outcomes of adipose-isolated MSCs appear to be less effective than the BM-MSC-based treatments [13, 15, 27–30].

In terms of availibility, AD-MSCs offer more abundant sources over BM-MSCs but lower amounts of stem cells among the nucleated cells. Adipose tissue isolation is more acceptable when compared to bone marrow extraction, even though liposuction or liposection both still require a surgical cut and a subcutaneous intervention. This creates a gap between the current trend of reducing animal stress in equine veterinary medicine and the golden standard invasive sampling routines. Inevitably, it also creates an unmet need for establishing and standardizing a minimally invasive harvesting procedure of MSCs in horses.

This study presents the least invasively available source of MSCs in horses to date: the outer root sheath of the mane hair follicle. The goal of the study was to develop MSC-relevant cell populations that could be harvested with the minimum invasiveness known, scalable to therapy-relevant numbers, and displaying features of MSCs equivalent to those harvested from the bone marrow and fat tissue. A further goal was to standardize the isolation and culture of these MSCs in order to qualify them as a fitting candidate for veterinary therapeutic requirements. To the best of our knowledge, an isolation and culture of mesenchymal stem cells from equine hair follicle has not been reported to this day, highlighting novelty and impact of the proposed evidence.

Application of animal stem cells for the reasons of tissue regeneration presents a minor sector of veterinary medicine, mostly for the reasons of cost-effectiveness. The disabling degenerative injuries typically result in euthanizing the injured animals. Quite differently, when it comes to improving the health condition of the running horse, both the cost effectiveness and the specific breeding context impose higher demands on the therapeutical methods. This paper provides a method for minimally invasive harvesting, isolation and culturing of autologous equine stem cells from hair follicle, which may in future be used for treating the potential equine diseases including lameness, particularly in the aforementioned context.

## Methods

### Specimen Selection and Sample Size

This study of isolating MSCs from hair follicles was approved by the Ethical Committee of Medical Faculty, University of Leipzig, Germany (ref. 427/16-ek, 27.02.2017). The horses included in this study all scored 5 according to the Henneke horse body condition scoring system [35]. Skin samples were collected 1 h *post mortem* from euthanized horses by the courtesy of the Institute of Veterinary Anatomy, University of Leipzig, Germany (*N* = 6, mean age 9.6 years). Fresh sample tissue of horse forehead skin and haunch adipose were harvested to isolate the hair follicle MSCs (eMSCORS) and horse adipose-derived MSCs (eADMSC) as a comparative control.

### Isolation of Horse Hair Follicles

**Fig. 1:**
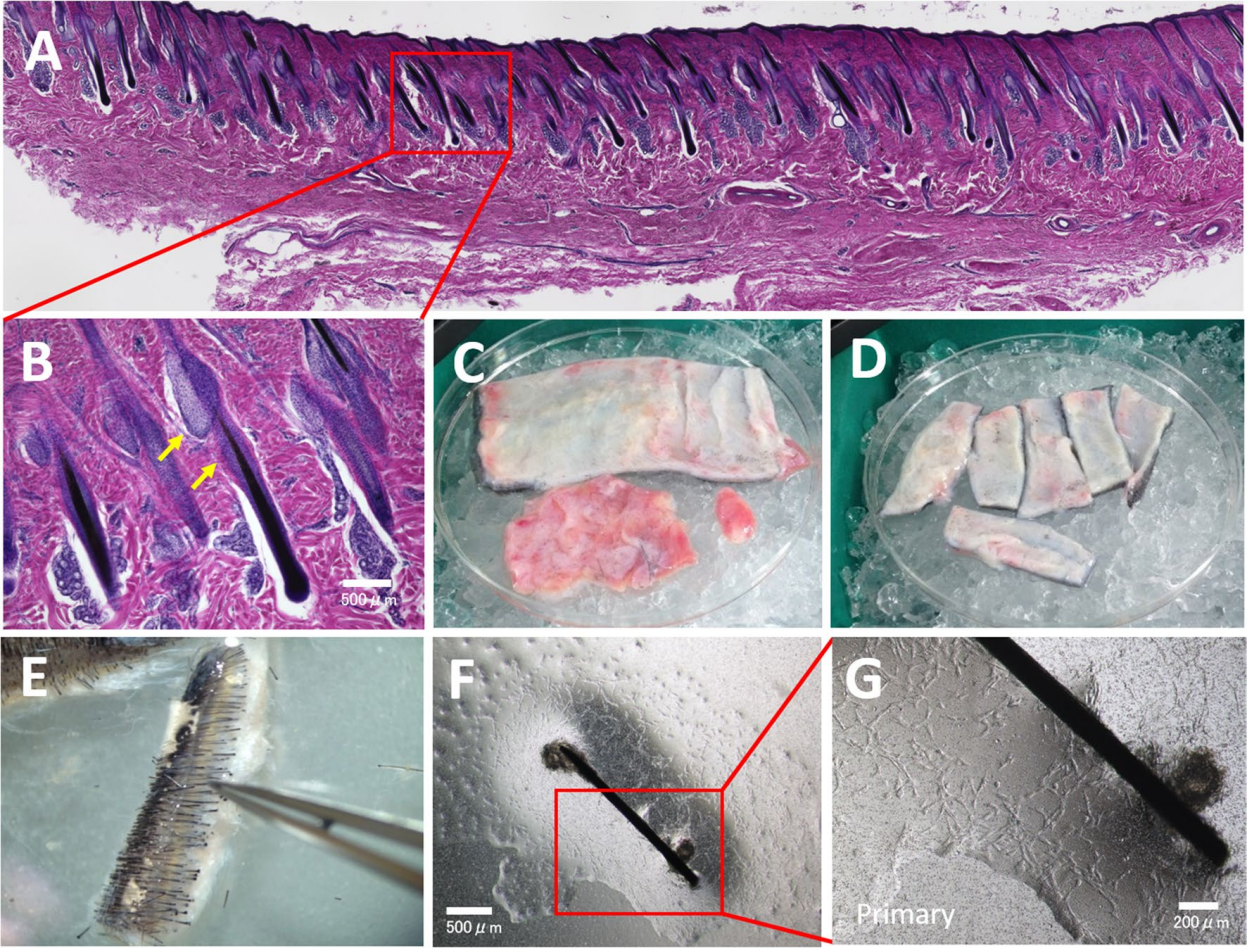
Histological structure of equine forehead skin and eMSCORS isolation using air-liquid interface method (*n* = 6). **A** Cross-section of forehead skin stained with H&E to study the anatomical structure of the equine donor skin. Sebaceous glands and the bulge region of the ORS were observed in the upper distal hair follicle in the dermis (**B**, yellow arrows). **C**, **D** A forehead skin (3 cm × 5 cm, large size for presentation) was dissected with its subcutaneous tissue removed, and sliced into long strips. **E** After collagenase treatment, the forehead skin dermis was loosened and hair follicles were plucked downwards from the side edge of the skin. **F**, **G** Hair follicles were transferred onto a Transwell membrane, and spindle-shaped cells migrated from the ORS onto the mesh, forming a cell layer; enlarged area of the ORS outgrowth, shown in G. Magnification: **A** a mosaic photo stitched from several photos in 4x by Keyence microscope, **B**, **D** 4x, **E**, **F**, **G** 10x

The hairs of the forehead area were shortened to a 2–3 mm length using an electric clipper. After hair removal, the foreskin area was rinsed with running water and disinfected using 70% ethanol. Full-thickness forehead skin was excised excluding subcutaneous tissue. The harvested forehead skin was preserved in the Dulbecco‘s Phosphate Buffer Saline (DPBS) containing 100 U/mL Penicillin/Streptomycin and 82.5 µg/mL Amphothericin B (ThermoFisher Scientific, Darmstadt, Germany) and brought to the laboratory. The forehead skin was rinsed thoroughly with ice cold DPBS containing antibiotics and cut into 5 mm x 30 mm rectangles in the direction of hair growth, to expose intact hair follicles. This step facilitated the following enzymatic treatment and follicle extraction. The rinsed skin fragments were incubated with 2 mg/ml Collagenase V (Sigma-Aldrich GmbH, Schnelldorf, Germany) at 37 °C for 5 h to loosen the tissue. The detachment of the dermis and dermal reticular fibers, as well as the hair follicle exposure from the dermis inner side was determined microscopically upon digestion. (Fig. 1B). Hair follicle plucking from the inner dermis side was achieved by clipping the hair shaft using pointy forceps, followed by repetitive pushing and pulling of the hair within the dermis to loosen the follicle from the connective tissue. Once loosened, the hair shaft exposed part underneath the skin was pulled out from the inner dermal side. Plucked hair follicles were harvested and preserved in DPBS with antibiotics for further cell isolation.

### Isolation of the Mesenchymal Stem Cells from the Hair Follicles

Mesenchymal stem cells (MSCs) were cultured from the isolated plucked equine hair follicles. Briefly, the proximal hair portion was excised, the follicles were inten-sively rinsed in DPBS-antibiotics solution, and treated with 5 mg/ml Collagenase V for 8 min at 37 °C to loosen the extracellular matrix of the Outer Root Sheath (ORS). After neutralization, horse hair follicles were transferred to Corning Transwell® mesh in a suspended well (0.4 μm pore diameter, 6-well plate template, Corning Inc., New York, NY, USA). The lower compartment was filled with 0.9 ml of eMSCORS Isolation Medium (Table 1). The primary culture was incubated in hypoxic conditions (5% O2, 5% CO2) at 37 °C.After 7 days of cultivation, the resulting cells called eMSCORS started to migrate from the ORS onto the Transwell mesh, underwent divisions and formed a monolayer, displaying a spindle-like and elongated morphology. After 17–24 days the cells reached ~ 70% confluency and were split using 0.05% Trypsin / 0.03% EDTA (ThermoFisher Scientific, Darmstadt, Germany), subcultured into a 6 well plate and left 24 h to adhere. Unattached cells were rinsed off, and the adherent ones were culti-vated in the eMSCORS Cultivation Medium under hypoxic conditions. The resulting eMSCORS were labelled as P0, and passaged upon reaching 90% confluence at a ratio of 1:3. eMSCORS in passages up to P5 were used for further characterization and differentiation experiments.

**Table 1:** Medium compositions

Washing Medium	DPBS, 100 U/ml Penicillin, 100 µg/ml Streptomycin, 82.5 µg/mL Amphotericin B
eMSCORS Isolation Medium	DMEM (Low Glucose), 10% Horse Serum, 1% ITS Premix, 40ng/ml bFGF, 20ng/ml rhEGF, 2mM L-Glutamine, Penicillin 100U/ml, Streptomycin 100 µg/ml
eMSCORS Cultivation Medium	DMEM (Low Glucose), 10% Horse Serum, 2mM L-Glutamine, Penicillin 100U/ml, Streptomycin 100 µg/ml
MSC Adipogenic Medium	DMEM (Low Glucose), 10% FBS, 1 µM dexamethasone, 500 µM IBMX, 100 µM indomethacin, 10 µM/mL insulin, 1% non-essential amino acids and 1% L-glutamine
MSC Chondrogenic Medium	DMEM (Low Glucose)/F12, 1% Human Serum, 1% ITS Premix, 2mM L-Glutamine, 10ng/ml TGF-β1, 10ng/ml BMP-4, 50ug/ml Ascorbic Acid, 50ug/ml Na Pyruvate, 1% Non-essential AA
MSC Osteogenic Medium	DMEM (Low Glucose), 10% Fetal Bovine Serum, 2mM L-Glutamine, 200nM Dexamethasone, 50ug/ml Ascorbic Acid, 10mM β-glycerophosphate

### Isolation of Mesenchymal Stem Cells from Adipose Tissue

To harvest the adipose tissue, hairs from the hip skin were shaved with electric clippers. The prepared area was then washed with detergent, rinsed with running water and disinfected with 70% ethanol. A 10 × 10 cm^2^ hip skin square was resected and withdrawn using forceps. The adipose tissue was excised into 1 cm x 3 cm x 2 cm pieces and collected in DPBS with antibiotics. Particular care was taken to maintain the muscle layer intact in order to avoid bleeding. Approximately 30 – 50 g of adipose tissue was collected, transferred to the laboratory and processed at room temperature to avoid fat solidification. The horse adipose tissue was thoroughly rinsed with DPBS with antibiotics, sliced into 1 mm3 pieces and incubated in 2 mg/ml Collagenase V at 37 °C for 4 h with intermittent shaking. After digestion, the tissue sample was layered ito an upper luminous yellow floating oil phase and an aqueous phase. The enzymatic digestion reaction was neutralized with 2 ml of FBS. The preparation was vigorously vortexed for 1 min to release all of the cells in the aqueous phase, and centrifuged for 10 min at 1600 g, room temperature. The cell pellet was rinsed twice in DPBS. The cell suspension was filtered through a 70 μm nylon mesh, seeded onto 6-well plates and cultured in eMSCORS Cultivation Medium. The medium was changed twice a week and cells were passaged upon reaching 90% confluence at 1:3 ratio. The resulting eADMSCs were used up to P5 for further characterization and differentiation experiments.

### Characterization of Cell Mobility

Six thousand cells of eqMSCORS from a single isolation were seeded in triplicate (*N* = 3) on 4-well chamber slide (ibidi GmbH, Planegg, Germany) and incubated for 24 h in hypoxic environment at 37 °C. The mobility of the cells was photo-documented by tracking 20 independent cells (*n* = 20) sequentially captured in a Keyence BZ-9000 Live Cell Imaging System (Keyence GmbH, Neu-Isenburg, DE, USA) at 10-min interval over a period of 24 h. In total, 146 images per track were imported as temporal stacks to the ImageJ version 1.53a software (https://imagej.nih.gov/ij/) and analyzed with the ImageJ Chemotaxis/Migration tool (https://ibidi.com/img/cms/products/software/chemotaxis_tool). Cell movement was quantified by manually tracking each of 20 cells per triplicate, by marking their position in each frame of the image stack. The Keyence-produced tracking files (tab-delimited text) were imported by the Chemotaxis and Migration tool in order to analyze cell size, accumulated and Euclidean distances, velocity, and directionality.

### Histological Sectioning and Staining of Horse Forehead Skin Tissue

Horse forehead skin was fixed in 10% formaldehyde solution, embedded in paraffin and sliced into 5 μm-thick sections. After de-paraffinization, the sections were stained with Hematoxylin and Eosin (H&E, Carl Roth GmbH, Karlsruhe, DE). The stained histological sections were documented under brightfield in objective magnification of 4x, 10x, 20x using Keyence BZ-9000 Fluorescence Microscope (Keyence GmbH, Neu-Isenburg, DE).

### Analysis of Cell Surface Marker Expression by Flow Cytometry

In order to investigate the surface marker profiles of the isolated equine cells, the expression of ISCT-defined MSC surface markers in both eMSCORS and eADMSC at passage 2 was analyzed by Flow Cytometry. CD29, CD44, and CD90 were used as positive markers, whereas CD14, CD34, and CD45 served as the exclusive negative markers. Information of antibodies and isotype controls are shown in Table 2. Fluorescence intensity of labelled cells was detected using BD FACS Canto II (BD Biosciences, San Jose, CA, USA), and the results were analyzed with FlowJo 10.0 software (BD Biosciences, San Jose, CA, USA).

**Table 2:** Antibody information

Antibody	Host species isotype	Clone	Reactivity	Company	Dilution
CD29-A488	Mouse IgG1	TS2/16	Anti-human	Biolegend, San Diego, CA	1:20
CD44-APC	Rat IgG2b	IM7	Anti-mouse	BD, Franklin Lakes, NJ	1:100
CD90-APC	Mouse IgG1	5E10	Anti-human	BD, Franklin Lakes, NJ	1:100
CD14-APC	Mouse IgG1	134,620	Anti-human	R&D, Minneapolis, MN	1:50
CD34-FITC	Mouse IgG3	43A1	Anti-human	AdipoGen Lifesciences, San Diego, CA	1:25
CD45-A488	Mouse IgG2a	F10-89-4	Anti-human	Bio-Rad, Hercules, CA	1:5
CD29-A488	Mouse IgG1k	isotype control	Anti-human	Biolegend, San Diego, CA	1:20
CD44-APC	Rat IgG2bk	isotype control	Anti-mouse	Biolegend, San Diego, CA	1:100
CD90-APC	Mouse IgG2aκ	isotype control	Anti-human	Biolegend, San Diego, CA	1:100
CD14-APC	Mouse IgG1κ	isotype control	Anti-human	Biolegend, San Diego, CA	1:50
CD34-FITC	Mouse IgG3κ	isotype control	Anti-human	Biolegend, San Diego, CA	1:25
CD45-A488	Mouse IgG2aκ	isotype control	Anti-human	Biolegend, San Diego, CA	1:5

### “Tri-Lineage” Differentiation of Obtained Cells

To study the differentiation potential of eMSCORS and eADMSCs towards mesodermal cell lines, these were differentiated in vitro under adipogenic, chondrogenic- and osteogenic-inducing culture conditions. Compositions of differentiation media are listed in Table 1. Briefly, for adipogenic induction, cells were plated at 6 × 103/cm2 density and cultured in adipogenic differentiation medium (Table 1) for 21 days in normoxic conditions (37 °C, 5% CO2). The resulting cells were analysed microscopically based on their morphology and stained with Oil Red O dye. Differentiation towards the chondrogenic lineage was achieved using 2.5 × 10^5^ eMSCORS centrifuged at 800 g to generate a high-density pellet culture. The pellets were further differentiated for 21 days in chondrogenic differentiation medium (Table 1). The pellets were sectioned and stained using H&E and Alcian Blue (Sigma-Aldrich Chemie GmbH, Steinheim, DE) to observe the chondrogenic morphology and stain proteoglycans, respectively. To achieve differentiation towards the osteogenic lineage, 2 × 10^4^/cm^2^ cells were cultured in osteogenic medium for 21 days. The deposition of calcium phosphate was detected by Alizarin Red staining, and the activity of Alkaline Phosphatase (ALP) was visualized using a chromogenic reaction with a nitro blue tetrazolium/ 5-bromo-4-chloro-3-indolyl phosphate (BCIP/NBT) substrate (Greiner Diagnostic GmbH, Bahlingen, DE). Images of the stained preparations were captured under brightfield at 4x, 10x, and 20x magnification using Keyence BZ-9000 Fluorescence Microscope (Keyence GmbH, Neu-Isenburg, DE).

## Results

### Isolation, Morphological Characterization and Migration Capacity Analysis of eMSCORS and eADMSC

The eMSCORS were isolated from the hair follicles of the equine forehead skin area, targeted as being rich with anagen hair. The skin specimen were histologically cross-sectioned and stained to study the cell donor site (Fig. 1A). The skin of the mane area displayed abundancy in dermal fibres (Fig. 1B) and hair follicles, with distinct sebaceous glands, sweat glands, dermal papilla and ORS. The bulge region of the ORS was located in the distal part of the hair follicles. 120 hair follicles were plucked from each skin specimen in order to isolate the eMSCORS.

**Fig. 2:**
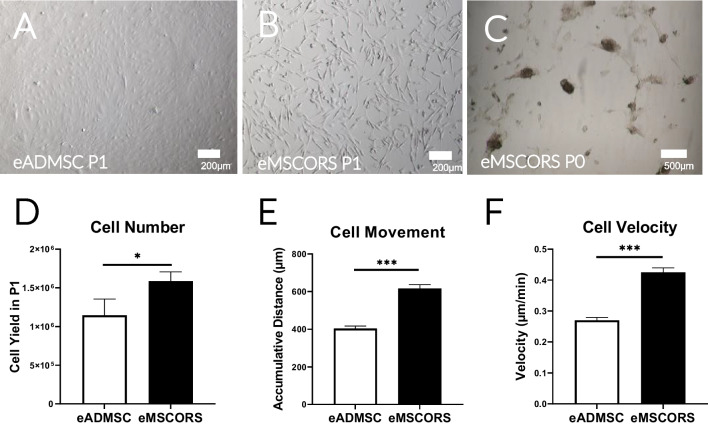
In vitro cultivation and cell mobility properties of eMSCORS and eADMSC (*n* = 3). **A** Confluent cell layer of eADMSC after being isolated and cultivated in P1. **B** Confluent cell layers of eMSCORS exhibited an elongated dendritic-like morphology in P1. **C** eMSCORS formed aggregated colonies after being subcultured from the Transwell meshes into cell culture flasks in P0. **D** Cell yields per isolation of eADMSC and eMSCORS at P1. **E**, **F** Euclidean distance and movement velocity of eADMSC and eMSCORS. Statistical significance: * *p* < 0.05, *** *p* < 0.001

Air-liquid interface conditions facilitated cell migration from the ORS onto the Transwell porous membrane. All hair follicles yielded cell outgrowth. The migrating cells displayed a dendritic-like morphology. They proliferated and formed an 80% confluent cell layer within 21 days of culture (Fig. 1D). The harvested eMSCORS easily adhered to the cell culture plastic and formed cell colonies (data not shown). eMSCORS proliferated rapidly in a cell doubling time of 1.02 days, yielding 1.59 ± 0.58 × 10^6^ cells in P1 upon isolation of 120 hair follicles. No morphological signs of cell senescence or apoptosis were observed until Passage 5 (data not shown). Adherent eADMSCs isolated from the hip adipose tissue recovered for 48 days and then proliferated. eADMSC divided rapidly with a cell doubling time of 1.18 days, which produced 1.14 ± 0.55 × 10^6^ cells in P0 upon each isolation of adipose tissue. eADMSCs acquired flattened and spindle shape, different from that of the eMSCORS (Fig. 2A-D).

eMSCORS showed high mobility in culture. Using time lapse imaging to analyze cell movement, eMSCORS migrated at 0.42 ± 0.025 μm/min mean velocity, reaching a total trajectory of 615.84 ± 35.2 μm as mean accumulated distance, and altering their nett position by migrating an effective Euclidian distance of 98.16 ± 15.85 μm (Fig. 1). In comparison, eADMSC showed lower cell motility than eMSCORS migrating at average velocity of 0.27 ± 0.12 μm/min and reaching a cumulative trajectory of 403.65 ± 180.52 μm. (*p* < 0.001, Fig. 2E-F).

These results indicated a successful isolation of eMSCORS using an air-liquid interface method, which yielded substantial amount of cells with a comparable population doubling time and a higher motility when compared with eADMSC.

### Phenotypic Characterization of eMSCORS and eADMSC

**Fig. 3:**
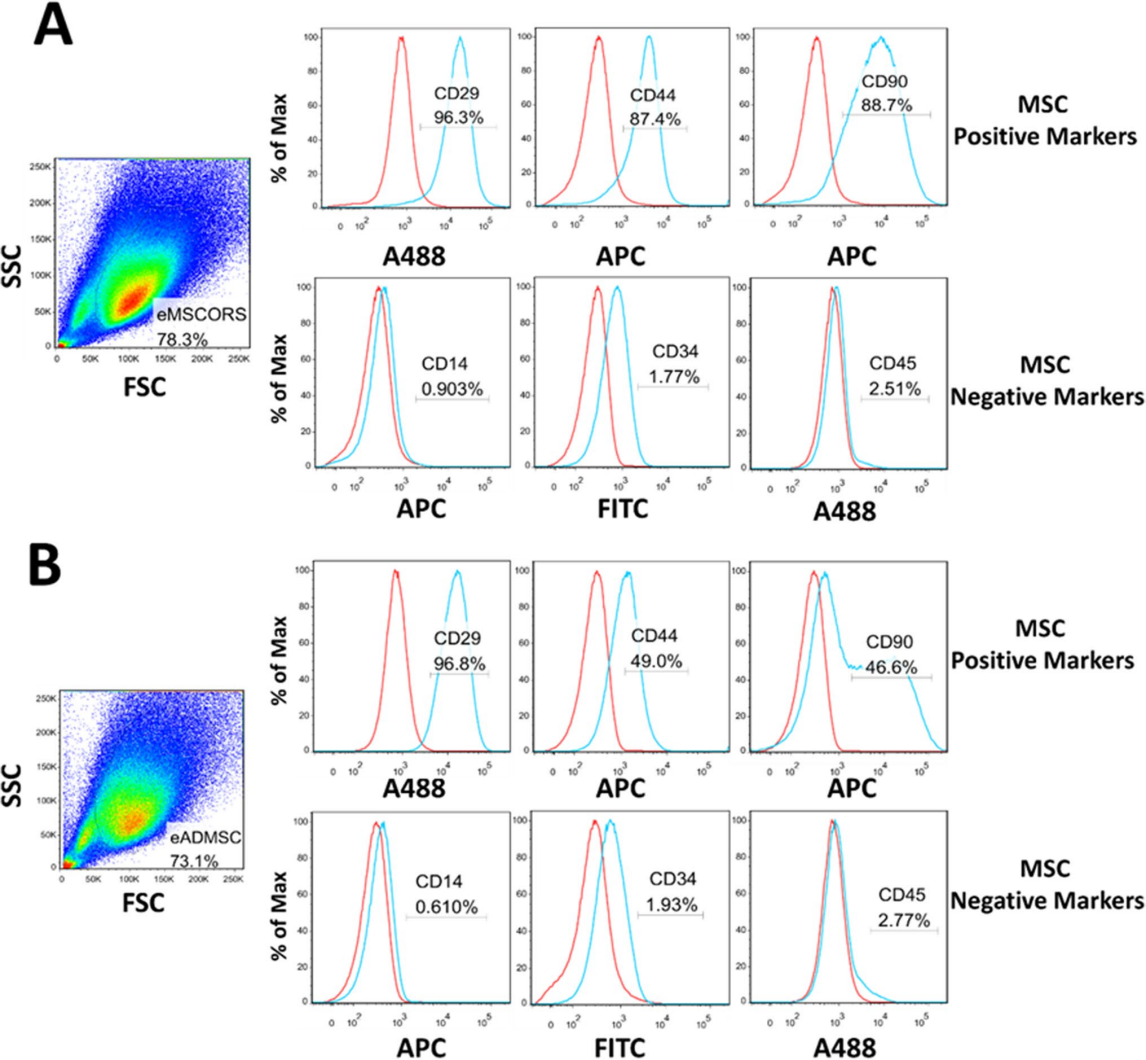
Analysis of MSC-related biomarkers expressed in horse cells using flow cytometry (*n* = 3). To characterize the phenotypes of isolated eMSCORS and eADMSCS, cells were stained with fluorescently-labeled antibodies against the surface markers, according to the MSC marker definition panel. Cell populations of eMSCORS and eADMSCS were displayed and gated in the plot graph of forward scatter (FSC) versus side scatter (SSC). The expression of MSC-positive markers (CD29, CD44, CD90) and negative markers (CD14, CD34, CD45) are indicated by fluorescence intensity (blue) against the isotype control (red). Representative plot graphs and histograms are shown for eMSCORS (**A**) and eADMSC (**B**)

Both eMSCORS and eADMSCS were phenotypically characterized for the expression of MSC-specific surface markers with Flow Cytometry. Anti-human antibodies successfully cross-reacted and labeled equine MSC surface markers (CD29, CD44, CD90), whilst not labeling the negative markers (CD14, CD34, CD45). Figure 3 displays the fluorescence intensity of each marker labeled with APC or FITC fluorescence against the isotype controls. eqMSCORS expressed higher levels of MSC-typical markers when compared to eADMSC: CD29 96.60% vs. 68.50%, CD44 86.50% vs. 50.60%, and CD90 86.00% vs. 52.1%, respectively. Cells expressing negative marker were identified as less than 2% of the total gated population.

### Tri-Lineage Differentiation of eMSCORS and eADMSCS

To evaluate the differentiation potentials of eqMSCORS and eADMSCS, both were subjected to chondro-, osteo- and adipogenic differentiation for 21 days.

**Fig. 4:**
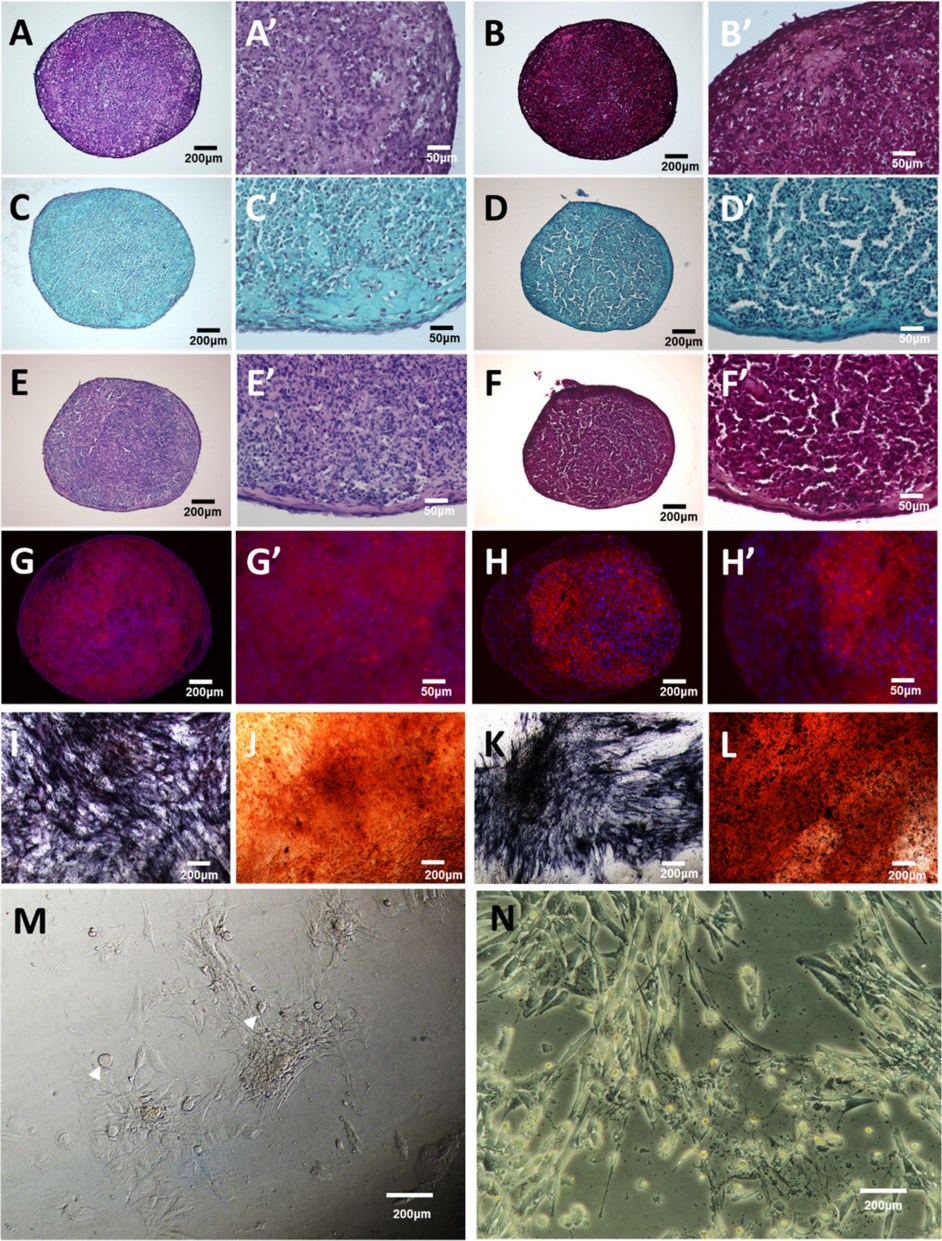
Tri-lineage differentiation of eMSCORS and eADMSCS (*n* = 3). **A-H** Chondrogenic differentiation was achieved after 3 weeks of pellet culture; the cartilaginous pellets of Emscors (A,C,E,G) and eADMSCS (B,D,F,H) were stained with H&E (**A**, **B**), Alcian Blue (**C, D**), Safranin O (**E, F**), and underwent immune-detection of collagen type II (**G**, **H**). Enlarged photos of eMSCORS with higher magnifications are shown in A’, C’, E’, G’, and eADMSC in B’, D’, F’, H’. Osteogenic differentiation was induced for 3 weeks; cells were stained with BCIP/NBT to show ALP activity (**I, K**), and stained with Alizarin Red (**J, L**) to detect calcium deposition in eMSCORS (I,J) and eADMSCS (**K, L**). After adipogenic differentiation, morphological changes were observed in eMSCORS (**M**, white arrows) and eADMSCS (**N**). Magnifications: (**A-M**) 10x, (**A’-H’**) 40x, (**N**) 20x

After being differentiated in high-mass pellet culture, both cell types showed deposition of a cartilaginous matrix consistent with differentiation towards the chondrogenic lineage, including proteoglycan identified by Alcian Blue and Safranin-O stainings, as well as Type II Collagen labeled by immunostaining, as shown in Fig. 4. Even though eMSCORS and eADMSCS pellet did not show significant differences in terms of pellet size or ECM deposition, they showed morphological differences. eMSCORS pellets were characterised as compact structures, whereas eADMSC pellets were in comparison more loose, with substantial structure gaps.

After 21 days of differentiation under osteoinducing conditions, both eqMSCORS and eADMSC deposited a considerable layer of calcium phosphate, as identified by intensive Alizarin Red staining. Alcaline phosphatase (ALP) activity was evaluated using BCIP/NBT substrate and high levels of dark brown catalysate were visualized intracellularly both in eMSCORS and eADMSCS.

Upon adipogenic differentiation, the cells altered their morphology and acquired an oval, compact shape, consistent with the typical morphology of adypocites. Intracellularly, perinuclear granules were formed. Oil Red staining failed to label the lipid vesicles.

## Discussion

This study reports a successful isolation, cultivation and characterization of MSCs from the follicles of equine mane hair that are easy to reproduce and robust in cell yield. The equine MSCs were isolated by the means of migration from the follicle outer root sheath in an air-liquid-interface experimental set up, by proliferation on the permeable membrane and transfer to adherent plastic surface. The method used recapitulates the previously postulated procedure for isolation of human MSCs from hair follicle [36], with the sole variation of using horse serum instead of FBS. As the equine follicle-derived MSCs were isolated from the outer root sheath, we accordingly named the equine MSCs: eMSCORS. They fulfilled the minimal requirements for characterizing human MSCs as defined by the International Society for Cell Therapy (ISCT) - adherence to plastic, proliferation, surface marker expression profile and differentiation potentials [37] The latter amounts to the previously reported differentiation propensity of freshly isolated equine bone marrow-derived MSCs (BM-MSCs) since BM-MSCs have been reported to fail at undergoing a trilineage differentiation [1, 38]. The eMSCORS, on the other hand, differentiated into osteogenic and chondrogenic lineage and fulfilled the criteria for reaching adipopocyte-like phenotype in terms of morphology and presence of perinuclear granules, while lacking the labelling of lipid vesicles by the Oil Red method, hereby matching the known differentiation capacity of BM-MSCs.

Intracellularly, eMSCORS expressed high levels of CD29, CD44 and CD90 taken for MSC positive markers, yet not expressed MSC negative markers including CD14, CD34, CD45. Despite a widespread use of MSCs from non-humans, there is still some lack of consensus on criteria for defining these cells [35, 39, 40]. Generally, MSCs display plastic adherence and trilineage differentiation capacity, while not expressing the same panel of surface antigens described for human MSCs. For example, most mammalian MSCs derived from various tissues display CD44 and CD29 expression [39, 41, 42], while the expression of other MSC markers such as CD90, CD73 and CD105 varies depending on species and strains [43]. Within the frame of this study, we adhered to the minimal characterization panel accepted by the International Society for Cell Therapy and showed that the eMSCORS fit well to this expression profile. The expression profiles of CD90 and CD44 within the major subpopulation of the heterogeneous population of the cells in passage 2 show that both surface markers were expressed in to a degree lower percentage of the cells. This was due to the lower affinity of the antibodies against the horse antigens. Namely, antibodies of different origins were used to detect the surface antigen in horses, including human and mouse antibodies. Therefore, anti-mouse-CD44 and anti-human-CD90 antibodies might show lower labelling efficiency than in human or in mouse.

For all above stated, we consider the eMSCORS a subject to a future refining, or revising of the MSC characterization in horses.

The high numbers of cultured eMSCORS in this study appear to solve the issue of the usually low fraction of MSCs obtained in BMMSC or ADMSC preparations. So far, the differences in therapeuthic activity of expanded equine MSCs versus point-of-care products remain unknown [44]. By all means, no known adverse effect speaks against the use of in vitro expanded MSCs. Notably, the expanded eMSCORS match equine MSCs from other sources such as adipose tissue, in terms of fullfilling the ISCT-defined criteria. In terms of isolation efficiency and scalability, taking in account standard 120 follicles sampled from the punch-biopsy and standard 5 g of fat tissue sampled by liposuction or liposection leaves us with an approximate 4:1 cell number ratio at the culture point of P1 (1.59 × 106 eMSCORS vs. 1.14 × 106 eADMSCs). Eventually, the synergy in gain through non-invasive sampling in minimal amounts and the high cell yield upon culturing make eMSCORS a very versatile choice of cells for equine therapeutic purposes [36].

Our reported isolation method and culture of eMSCORS postulates the obtaining, culture and basic characterization of eqMSCORS. At the same time, several issues of therapeutical use of MSC use in equine veterinary medicine apply to the eMSCORS as well in terms of defined immunemodulatory effect, invasiveness, safety issues, and even production efficiency.

MSCs are widely used to mitigate inflammatory disorders given their immunomodulatory and paracrine effects, as well as their implication in direct tissue replacement, therefore providing a stable and regeneration-permissive microenvironment and taking part in its repair [7, 35, 45–48]. Equine MSC ability to induce endogenous MSC proliferation, chemotaxis, and paracrine response has been previously reported [44]. This is in accordance with the immunomodulatory effects observed in human and mouse MSCs from hair follicles (moMSCORS) [49, 50]. Although reported in MSCORS of two species, and cross-species, the potential of eMSCORS for immune modulation has yet to be shown before seeing them as potentially applicable in the treatment of inflammatory disorders. In particular, the eMSCORS cell yield could still be increased and their immunological effect yet needs to be documented.

Safety is an issue in all known stem cell therapies, but to the least extent in case of MSCs. Their main competitors among other horse stem cells, the iPSCs, carry a burden of maligant prospects, which can impair the treatment outcome [51–53] Use of adult MSCs, which are cautiously but not exclusively regarded as non-malignant, helps bypass these uncertainties [5, 54, 55]. The potential immunological issues concerning eMSCORS are expected to be non-substantial. Due to their lack of expression of CD40, CD80, and CD86 costimulatory molecules in basal conditions, MSCs are considered immune-privileged and therefore not prone to provoking an imune response in heterologous contexts [56, 57]. Yet, heterologous transplantation alows for donor-to-recipient transmission of infectious diseases. Therefore, the possibly safest and simplest way to treat a horse with MSCs is to use autologous cells. It is widely known that the least invasive harvesting method tends to be the safest too, hence all of the above again emphasizes the autologous hair follicle as a robust source of MSC in horse.

Besides, equine veterinary medicine routines do not enlist an absolutely non-invasive sampling of biological material for MSC isolation. The isolation of eMSCORS involves the sampling of the horse mane follicles by a minimal punch biopsy from the mane skin area. This procedure does leave room for improvement towards a completely non-invasive sampling, since no matter how small the diameter, the punch biopsy is still of invasive nature. Nevertheless, it is still way less invasive than any other known method for sampling MSCs from a live animal. The main advantage of the method of culturing eMSCORS from equine hair follicle is that it can be sampled with minimum invasivity and easily be applied autologously to a living animal. The risks arising from the sampling procedure are marginal. The sampling area is locally disinfected and anesthetised about 30–40 min before biopsy. A punch biopsy needle is used to create a 4 mm in diameter circular incision and hereby extract a small piece of skin. The degree of invasivity is determined by the degree of discomfort, which is minimal and comparable to that of taking a blood sample. The puncture inflicted by the punch biopsy is negligible and requires a single stich. By the means of stiching and disinfecting prior to biopsy, the procedure is safe from infection.

When considering therapeutic MSC applications in horses, particularly for addressing the most frequent procedure of tendon therapy in competitive running, the treatment objectives are further complicated by implications of breeding, training, healthcare and financial investment. Furthermore, personal effort and emotional aspects of animal bonding characterize race horse breeding as far more intricated than in farm-based breeding. Therefore, running horses present a potential high-end treatment group and also impose a special need for minimizing the stress and personalizing the therapeuthic procedures. Clearly, starting with this target group, there is a future benefit for the eMSCORS for equine regenerative medicine. Their use would help overcome the invasive nature of the conservative harvesting methods for MSCs such as liposection, lipoaspiration or bone marrow. Main obstacle to the use of eMSCORS is not the lack of their therapeutic benefit, but a general lack of interest to introduce such an application into the general clinical context of veterinary medicine.

Noteworthy, horse stem cell therapies appear to be lagging behind analog treatments for humans, especially in terms of meeting regulatory demands. Production processes for cell-based therapies in horses can at times qualify as point-of-care solutions. However, given the involvement of substantial manipulation methods, this often results in requirements to undergo ATMP-related regulations. Stem cell-based therapies in horses are to an extent less stringently defined than their human analogues. They are currently based on guidelines for animal cell-based products released by FDA in 2015 [58, 59] and the following guidelines issued by EMA in 2016 [60]. The lack of clearly defined regulatory requirements for therapeuthic products can be by-passed by a premature application within a frame of isolated investigative and healing effort so called clinical exemptions, which are nevertheless often ill-defined. The regulatory niche for it exists in terms of local manufacturing of ATMPs within an academic institution in Regulation (EU) 1394/2007 under the term “Hospital Exemption” [61].

Among the first solutions for improving such personalized strategies, especially the profilactic ones, biobanking of equine MSCs comes to mind since it holds a promise of making equine MSCs quickly available at any time point. Again, ideally the deposition of autologous, non-invasively obtained MSCs is clearly embodied in eqMSCORS. This would offer a less painful, safe foregoing for both acute and chronic treatments of horses, and particularly useful for fast addressing in a context of running injury treatment [26, 38, 44, 62].

For all the above-mentioned reasons, the eMSCORS currently present an optimal choice of non-invasively, abundantly, reproducibly attainable, safe cells for purposes of personalized therapies in equine veterinary medicine. Together with a translational therapeutic track, this study also advances basic knowledge of equine-based models for cartilage injuries, osteoarthritis and tendon and ligament injuries, similar to those observed in human [63–70]. Therefore, eqMSCORS are eligible for use in non-invasively based autologous or heterologous regenerative cell therapy models that could help understand and predict analogue outcomes in a number of human disorders and injuries.

## Data Availability

The data generated during the current study are available from the corresponding author on reasonable request.
